# First anatomical network analysis of fore- and hindlimb musculoskeletal modularity in bonobos, common chimpanzees, and humans

**DOI:** 10.1038/s41598-018-25262-6

**Published:** 2018-05-02

**Authors:** Rui Diogo, Julia L. Molnar, Campbell Rolian, Borja Esteve-Altava

**Affiliations:** 1 Department of Anatomy, Howard University College of Medicine, Washington DC, USA; 2 Department of comparative biology and experimental medicine, Faculty of Veterinary Medicine, University of Calgary, Calgary, Canada; 3 Structure & Motion Lab, Department of Comparative Biomedical Sciences, Royal Veterinary College, London, UK

## Abstract

Studies of morphological integration and modularity, and of anatomical complexity in human evolution typically focus on skeletal tissues. Here we provide the first network analysis of the musculoskeletal anatomy of both the fore- and hindlimbs of the two species of chimpanzee and humans. Contra long-accepted ideas, network analysis reveals that the hindlimb displays a pattern opposite to that of the forelimb: *Pan* big toe is typically seen as more independently mobile, but humans are actually the ones that have a separate module exclusively related to its movements. Different fore- vs hindlimb patterns are also seen for anatomical network complexity (i.e., complexity in the arrangement of bones and muscles). For instance, the human hindlimb is as complex as that of chimpanzees but the human forelimb is less complex than in *Pan*. Importantly, in contrast to the analysis of morphological integration using morphometric approaches, network analyses do not support the prediction that forelimb and hindlimb are more dissimilar in species with functionally divergent limbs such as bipedal humans.

## Introduction

Primate limbs are morphologically diverse, and much of this variation in musculoskeletal anatomy correlates with locomotor function. For example, arboreal and terrestrial quadrupeds (e.g., cebids, cercopithecoids) have limbs that are about equal in length, while forelimb-dominated taxa such as gibbons and spider monkeys have relatively longer forelimbs^1^. Similarly, species in which the hindlimb takes on a major role in propulsion, such as leapers (e.g., lemurs, tarsiers) and bipedally-committed humans, have relatively longer hindlimbs. Such morphological specializations are usually assumed to reflect an adaptive evolution in the context of a taxon’s locomotor ecology. At the same time, however, even disparately proportioned fore- and hindlimbs share the vast majority of their genetic and developmental architecture^2,3^, and this may cause the limbs to co-evolve despite divergent functions^4^. Therefore, in addition to external/ecological pressures, development may constrain the evolution of limb musculoskeletal anatomy. This potential conflict between selective pressures for locomotor specializations and evolutionary constraints imposed by a shared development has major implications for understanding evolutionary and developmental patterns in human evolution.

To date, however, studies of modularity and integration in primate limbs have focused exclusively on skeletal data, using morphometric and evolutionary quantitative genetics tools to study phenotypic (co)variation in continuous traits such as limb bone length e.g.^4–11^. These studies have suggested, for instance, that when fore- and hindlimbs perform similar functions in support and propulsion, they have a higher inter-limb integration, and co-vary strongly in size and shape^6,9,10^. In contrast, in species where the limbs have become specialized (e.g., humans, gibbons), co-variation among topologically similar elements of the fore- and hindlimb is substantially lower, i.e., these limbs have a lower inter-limb integration. This suggests that selection for functional specialization can reduce developmentally-based co-variation^6,10^.

In contrast, modularity and integration among other features of the limb musculoskeletal system, including for example the presence/absence of muscles, and the physical connections among bones and muscles, have yet to be comprehensively investigated, in part because these features are not amenable to the same types of quantitative analyses as morphometric traits. In the past few years, anatomical network analysis (AnNA) has enabled the study of connectivity patterns (e.g., bone-bone and bone-muscle connections) in a quantitative way, by using mathematical tools borrowed from network theory. AnNA has been used to test specific hypotheses about the evolution of morphological organization and modularity e.g.^12–15^. Importantly, AnNA uses topological organization and connectivity relationships (e.g., articulations and attachments) between anatomical structures and/or types of tissues (e.g., bones and muscles) in a way that can complement patterns gleaned from morphometric analysis of size and shape.

Until recently, detailed data on the soft tissues of our closest living relatives, chimpanzees (genus *Pan*), were only available from a few studies restricted to one or few individual(s), and/or to single anatomical regions (for bonobos, see, e.g.^16^). To fill this gap, we recently published studies which compiled all the available data on common chimpanzee and bonobo soft tissues, adding new information based on the dissection of numerous common chimpanzees^17,18^, as well as comprehensive musculoskeletal photographic atlases of common *chimpanzees and bonobos*^19,20^. When studied with AnNA^13,21–24^, these data open several avenues of research in evolutionary biology and biological anthropology, including investigations about the links between phenotype and genotype. Here we provide the first comparative anatomical network analysis of the musculoskeletal system of both the fore- and hindlimbs of modern humans, bonobos, and common chimpanzees. We tested three hypotheses on the patterns and processes of human limb evolution:

*H1) In humans and chimpanzees, complexity in bone-bone and muscle-bone network connectivity in the fore- or hindlimbs correlates with the functional complexity of that specific limb*. In *Pan*, the hindlimb serves functions in different types of locomotion (e.g., terrestrial bipedalism, vertical climbing) and in gross manipulative activities, including grasping branches. In contrast, human hindlimbs are mainly involved in terrestrial propulsion and have lost the ability to perform functions such as grasping. We thus predict that human hindlimbs will have a lower network connectivity than those of *Pan* because they have a lower functional complexity.

*H2) The evolutionary increase in the number of unique forearm muscles in human evolution*^17,18^ *resulted in a lower average number of muscle-bone connections per structure and therefore in lower anatomical complexity of our forelimbs compared to those of chimpanzees and bonobos*. This prediction comes from our previous AnNA of various tetrapod taxa, which have shown that in general an increase in the number of musculoskeletal structures leads to a decrease in the average number of connections per structure and thus of network complexity. For instance, the trend towards a decrease in the number of skull bones in tetrapod evolution (Williston’s Law) has been shown to have actually led to an increase in the density of connections and thus of morphological network complexity in *H*. *sapiens* (e.g.^12–14^). In AnNA, density of connections (*D*) is often used as a proxy for the complexity of the network because the number of functional possibilities and potential functional outcomes increases with the number of connections among parts (for more details, see Materials & Methods). So, for example, when two bones become fused in evolution the connections previously displayed by each bone, including between themselves, are now displayed by the single (fused) bone, increasing the average bone-bone connectivity density overall. H2 thus refers to whether such a negative correlation between the number of structures vs. the network density and complexity as seen in the evolution of the tetrapod skull also applies to the forearm muscles and thus to musculoskeletal systems as a whole. That is, we predict that similarly to the negative correlation seen in the evolution of the tetrapod skull *(i.e., fewer bones -* > *increase of average bone-bone network density and complexity)*, there is a negative correlation resulting from the increase in the number of forearm muscles in human evolution *(i.e., more muscles and musculoskeletal structures in total–* > *decrease of the average bone-muscle network density and complexity)*.

*H3) Anatomical integration between fore- and hindlimbs correlates with degree of shared function between these two limbs*. Specifically, we predict that in the bipedal humans, in which the forelimb and hindlimb are less functionally convergent than they are in *Pan*, the similarity in network structure between the two types of limbs will also be lower.

## Network parameters of fore- and hindlimbs

Our network analyses reveal that the musculoskeletal networks in the forelimbs of *Pan troglodytes* and *Pan paniscus* are more complex (higher D and C, lower L) and less anisomeric (lower H) than the forelimb of *Homo sapiens* (Table 1; SI1 Tables 1, 2). Moreover, the forelimbs of *Pan troglodytes* and *Pan paniscus* are more similar to each other (FL Average Relative Difference 5.3%; SI1 Table 2) than they are to *Homo sapiens* (FL-ARD 11.3% and 7.5%, respectively). In contrast, the network parameters are more ambiguous with respect to network complexity of hindlimbs (Table 1; SI1 Tables 1 and 3). The values for connective density (D) and path lengths (L) suggest that the hindlimb of *Homo sapiens* is more complex than *Pan*, but clustering complexity (C) values contradict this pattern. Regarding similarity, the hindlimbs of *Pan troglodytes* and *Pan paniscus* are more similar (HL-ARD 1.3%; SI1 Table 3) than they are to *Homo sapiens* (HL-ARD 4.7% and 5.8%, respectively). Last, regarding the forelimb-hindlimb similarity (hypothesis H3), *Homo sapiens* and *Pan paniscus* have an almost identical degree of inter-limb similarity (FL/HL-ARD 5.2% and 5.3%, respectively), whereas *Pan troglodytes* has a higher disparity between forelimb and hindlimb (FL/HL-ARD 8.1%) (SI1 Table 4). At the skeletal level the three species have the same network configuration for forelimbs and hindlimbs, respectively (SITable 12). Thus, no species has a more or less complex forelimb or hindlimb than another regarding topology (all FL- and HL-ARD 0%; SI1 Tables 13 and 14). Last, the forelimb-hindlimb similarity of the skeletal component is the same in the three species (FL/HL-ARD 7.6%; SI1 Table 15).

**Table 1:** Network parameters of musculoskeletal network.

	N	K	D	C	L	H
*Homo sapiens* forelimb	94	193	0.044	0.380	3.10	0.923
*Pan troglodytes* forelimb	99	245	0.051	0.405	3.313	0.788
*Pan paniscus* forelimb	95	217	0.049	0.423	3.296	0.845
*Homo sapiens* hindlimb	91	207	0.051	0.378	3.222	0.909
*Pan troglodytes* hindlimb	97	217	0.047	0.381	3.320	0.962
*Pan paniscus* hindlimb	98	222	0.047	0.391	3.305	0.974

Regarding complexity (measured by the proxies D, C, L) of forelimbs vs. hindlimbs, the forelimbs are more complex in general at the musculoskeletal level than are the hindlimbs in *Pan troglodytes* and *Pan paniscus*, while in *Homo sapiens* the complexity is higher in the hindlimb (SI1 Table 15**)**. At the skeletal level results are not conclusive, the values of C indicating that the forelimb is more complex than the hindlimb, but the values of D and L suggesting otherwise. This means that the bones of the forelimb are more inter-connected (i.e., with more triangular-loops, C, which is often related to higher local integration of parts^13^) than the bones of the hindlimb. If we focus on the total density of connections (D) and their consequences on the effective proximity of parts (L), then the hindlimb skeleton is more complex because there are more connections among parts that bring distant elements closer, which may facilitate their integration as well^13^. This suggest that forelimbs and hindlimbs achieve anatomical integration via different organization strategies, in a case of evolutionary mosaicism. The differences between the forelimb and hindlimb autopod, in terms of topological organization and fusion among elements may explain these differences (e.g., the hand as more bones, and is thus more inter-connected than the foot).

## Connectivity modules of fore- and hindlimbs

**Figure 1:**
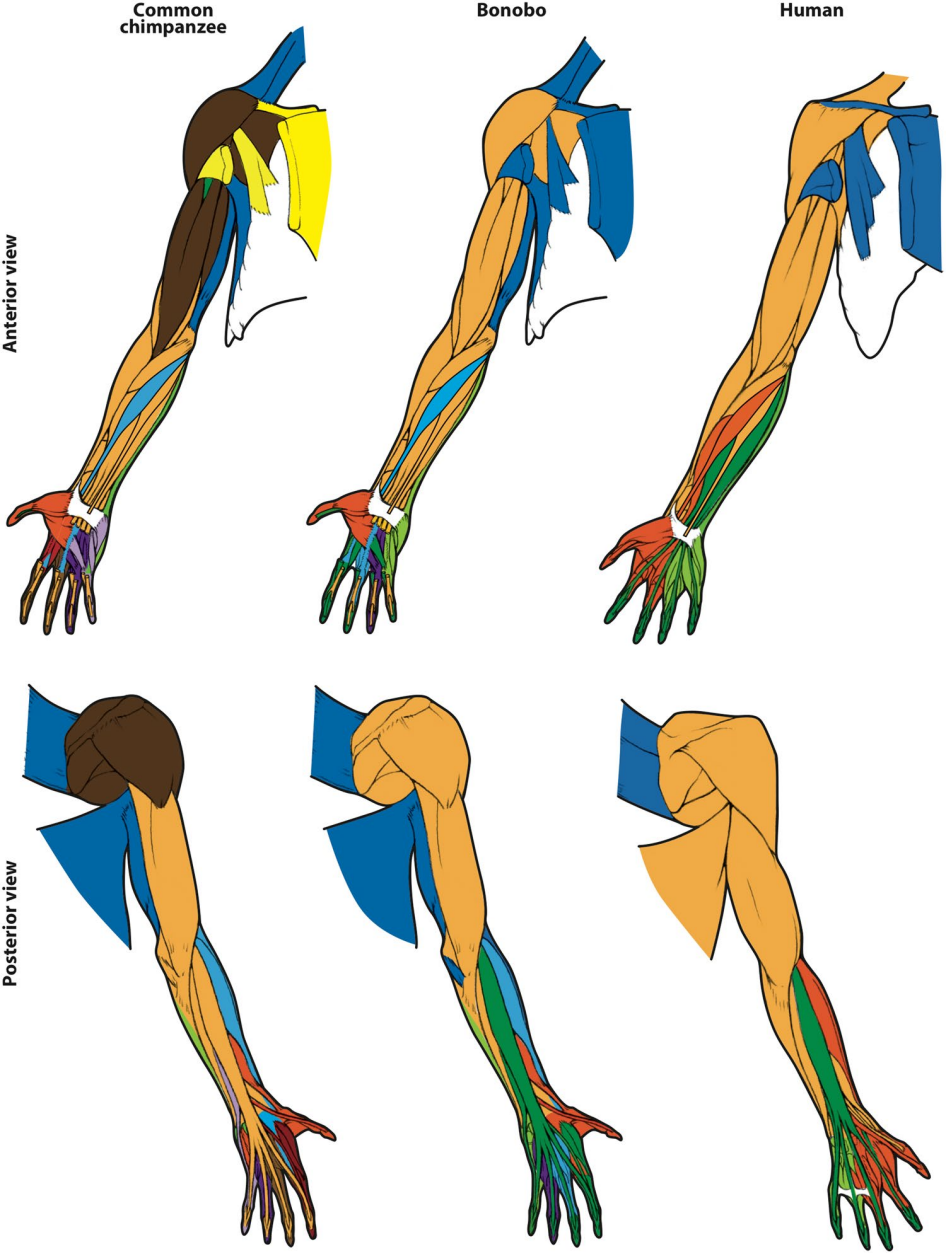
Forelimb anatomical network modules of common chimpanzees, bonobos and humans. While in humans there are only two modules related to the movements of the digits (dark orange: of digits 1–3, light green: of digits 4–5), in chimpanzees there are many more: four (dark orange: of thumb; light blue: of digits 2–3; magenta: of digit 4; light green: of digit 5) in bonobos, and six (dark orange: of thumb; red: of digit 2; brown: of digit 3; dark magenta: of digit 4; light green and dark blue: of digit 5) in common chimpanzees. For more details and for names and specific composition of each module, see text and SI1; for a list of all musculoskeletal structures and their connections, see SI2.

**Figure 2:**
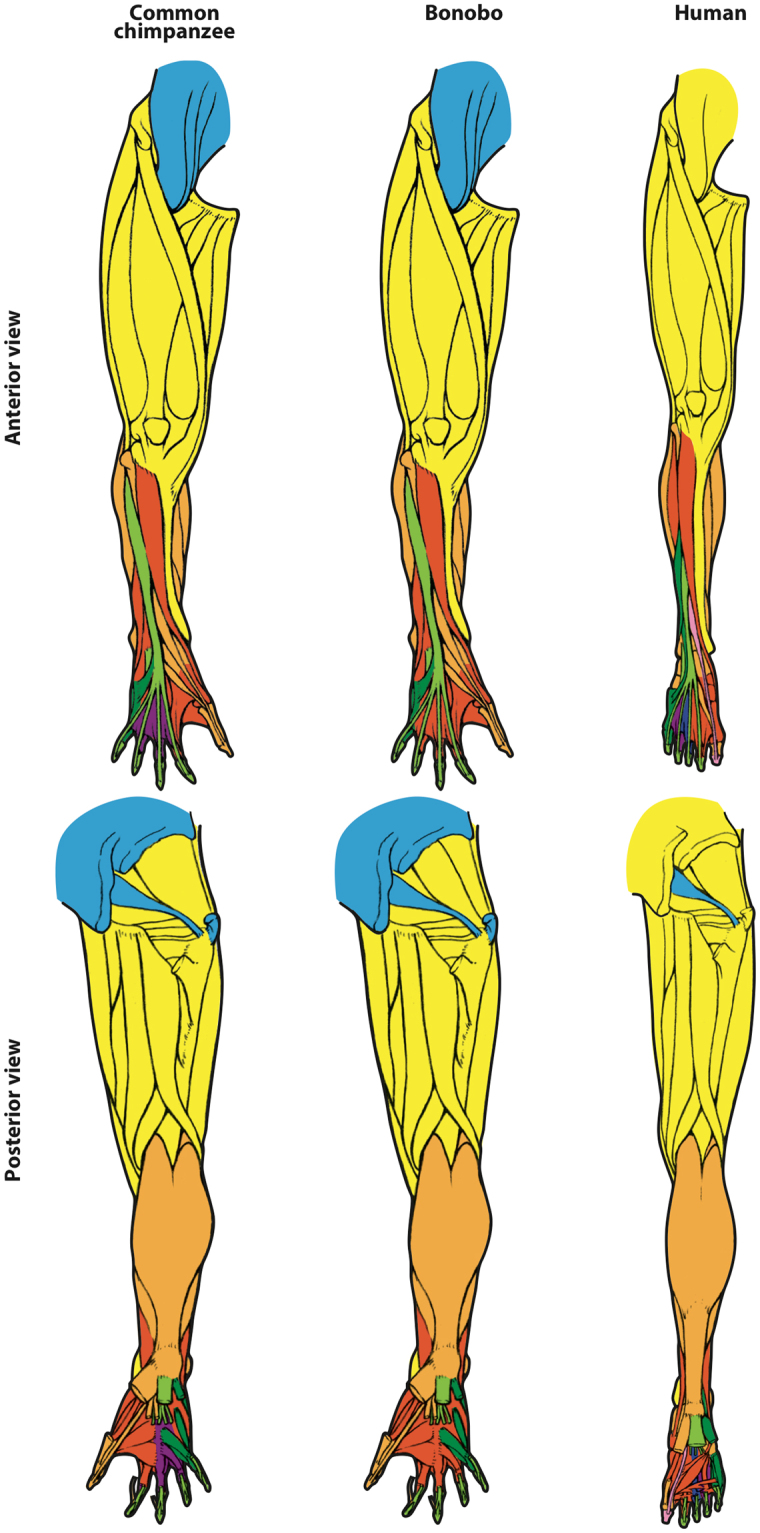
Hindlimb anatomical network modules of common chimpanzees, bonobos and humans. While in bonobos there is a single module related specifically to the movements of digits (dark green: of digit 5) and in common chimpanzees there are only two (dark green: of digit 5; magenta: of digits 3–4) in humans there are four, including one (pink) exclusively for the movement of the distal phalanx of the big toe (the other three are magenta: of digit 4; dark blue: of digit 3; dark green: of digit 5). For more details and for names and specific composition of each module, see text and SI1; for a list of all musculoskeletal structures and their connections, see SI2.

Among the specific connectivity modules obtained in our network analysis, we will focus on the modules that include both muscles and bones (Figs 1 and 2; SI1 Tables 5–11), which we can discuss in a more comprehensive functional and evolutionary context (complete skeletal modules are listed in SI1 Tables 16–22). The number of forelimb modules is remarkably different among bonobos, common chimpanzees, and humans (SI1 Table 5), but a detailed examination clearly shows that the *Pan* species share a more similar pattern (Fig. 1, SI1 Tables 6–8). For instance, while in humans there are only two modules related to the movements of the digits (of digits 1–3, and of digits 4–5, respectively), there are four in common chimpanzees (of thumb, of digits 2–3, of digit 4, and of digit 5) and six in bonobos (one for each digit plus an additional one for digit 5).

**Figure 3:**
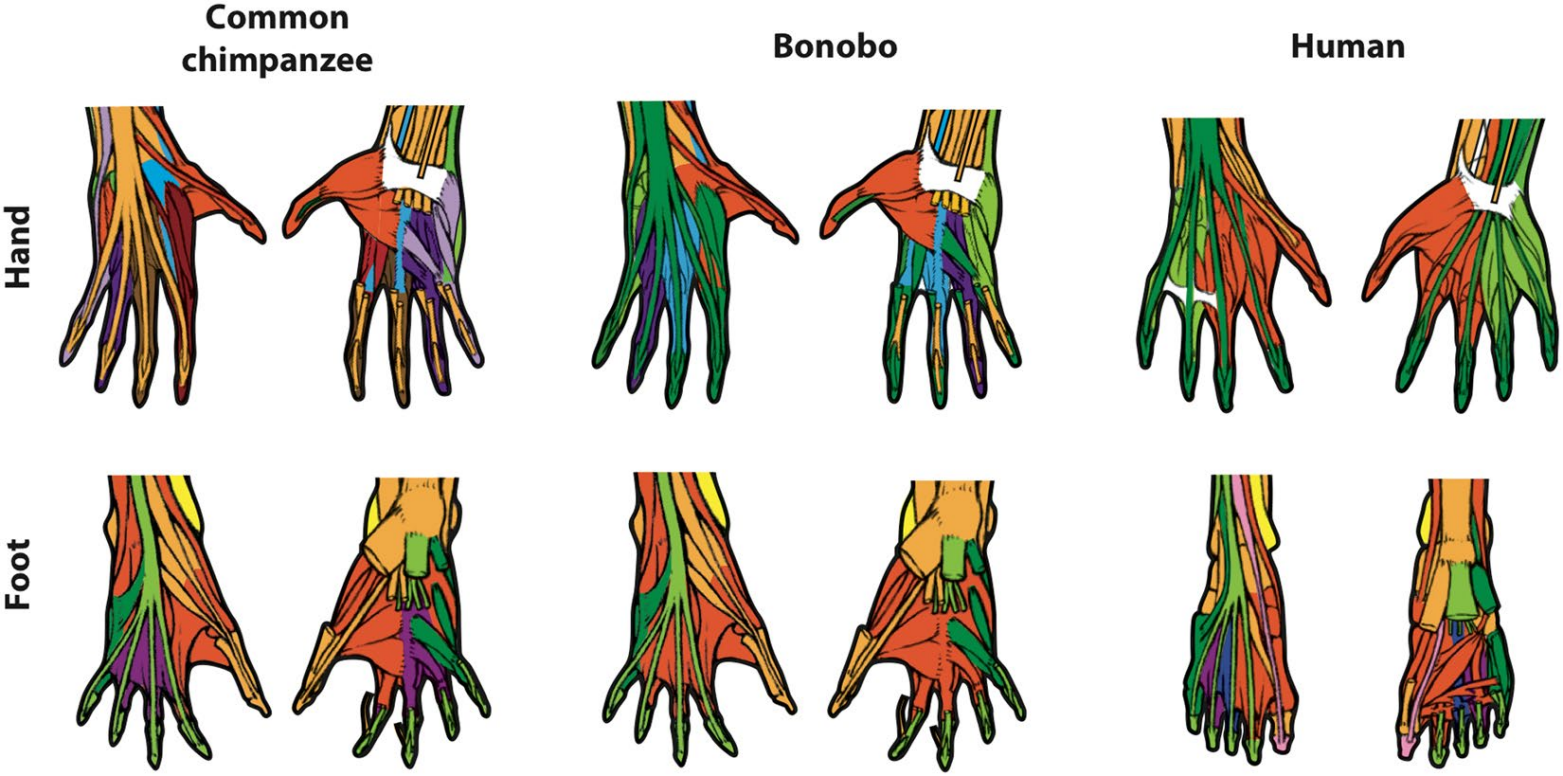
Details of hindlimb and forelimb autopod anatomical network modules of common chimpanzees, bonobos and humans. For more details and for names and specific composition of each module, see captions of Figs 1 and 2, text and SI1; for a list of all musculoskeletal structures and their connections, see SI2.

It has been argued that human evolution was linked to a loss of mobility of the individual digits other than the thumb, because humans have lost many of the serial hand muscles related to the movement of each other digit, such as the contrahentes (anisomerism: refs^25,26^). However, others have argued that the evolution of our ability to use and manufacture tools was also linked to an increase in modularity of the musculoskeletal structures of the thumb and/or related to its movements (reviewed in^27^). In addition, in a recent paper we have shown that humans do not have a separate thumb module^28^ in contrast to *Pan* and various other primates. Although this pattern might at first seem paradoxical, it is in fact easy to explain in view of our results. In *Pan* and other primates, the presence of serial muscles related to the movement of each non-thumb digit likely results - even if merely as a byproduct of this pattern - in a configuration in which each digit forms a separate module and thus in which the thumb does not share a module with any other digit (Figs 1 and 3, SI1 Tables 6–8). It is also possible that increased integration of the first three digits in humans is related functionally to the requirements of tool use and manufacture, and in particular, to the ability of these three digits, their muscles, and their associated carpal bones, to produce the muscular forces, and similarly withstand the large joint reaction forces associated with forceful precision grips, such as the three-jaw chuck^8,29,30^. In this sense, the results of the present work are particularly interesting, because the opposite pattern is observed in the hindlimb, in a striking example of mosaic evolution of the fore- vs. hindlimbs. Although the big toe is much more mobile in chimpanzees, only humans have a separate module exclusively related to the movements of digit 1, and separate modules for the movements the other digits (Figs 2 and 3, SI1 Tables 9–11). This individuation of the hallux in humans may relate to functional factors, for example, the greater amount of weight transfer through the first digit during stance in bipedal locomotion.

## Evolution of fore- and hindlimbs

In the Introduction we raised three major questions with implications for our understanding of hominin postcranial evolution. The first concerns the evolution of human bipedalism and complexity: H1) does the complexity in bone-bone and muscle-bone connectivity in the fore- vs hindlimb correlate with the functional complexity of that specific limb, which we predicted would result in decreased network complexity of our hindlimbs compared to those of chimpanzees? Primate limbs are usually “multi-purpose” structures that function differently in different ecological contexts^1^. For example, many Old World monkeys spend time in both terrestrial and arboreal environments, where interactions with the substrate vary considerably with respect to propulsion, grasping, and support. In contrast, human hindlimbs are entirely committed to bipedal support and propulsion. Following the reasoning that more complexity in locomotor behavior may lead to less constraints in the evolution of limb structures, we would predict that species in which a limb (fore- or hindlimb) is used in many locomotor/ecological contexts (i.e., is more behaviorally complex), that limb has a greater anatomical complexity of its musculoskeletal system, as indicated by network proxies. This would reflect a need to maintain a more versatile, generalized, and thus likely less constrained, anatomy compared to a highly specialized limb, such as the gibbon forelimb or the human hindlimb. Our results do not support this hypothesis. With respect to their musculoskeletal network organization, the hindlimbs of humans do not show lower complexity than those of chimpanzees: as noted above, only the values of C support this hypothesis, while the values of D and L actually suggest that human hindlimb is more complex that of the two species of chimps (Table 1; SI1 Table 1).

The second question concerns the evolution of the human forelimb, thumb mobility, and complexity: H2) during human evolution, was the increase in forearm muscle number accompanied by a decrease in the average number of muscle-bone connections per structure, resulting in decreased network complexity of our forelimbs compared to those of *Pan*? The only forelimb group for which humans have more muscles than most other extant primates are forearm muscles; this is because humans have two peculiar muscles associated with thumb movements - *extensor pollicis brevis* and *flexor pollicis longus* - that are absent in almost all extant tetrapods^17,18,27^. The presence of these two muscles in humans is consistent with the hypothesis that specialized thumb movements were important during human evolution, possibly related to stone tool manufacture and use, as noted above. Interestingly, the acquisition of these two extrinsic “thumb” muscles was accompanied by an evolutionary trend to lose both the hand muscles and forearm muscle tendons that attach to the other (2 to 5) digits, as also noted above^27^. One could thus predict that a network analysis would quantitatively show that an increase of forearm muscle number was accompanied by a decrease in the number of muscle-bone connections *(more muscles–* > *fewer musculoskeletal connections, thus less network complexity)*. In this case, the results of our network analysis do support this idea. That is, at a musculoskeletal level the values of network parameters show that the complexity in *Homo sapiens* forelimb is lower than in *Pan troglodytes* and *Pan paniscus* (lower D and C, higher L; Table 1; SI1 Table 1). Because at the skeletal level the forelimbs of the three species have the same topological organization, this suggest that the decrease of human forelimb musculoskeletal complexity has in fact involved only the interactions between muscles and bones.

The third question concerns the evolution of bipedalism and the co-evolution of fore- and hindlimbs: H3) does the inter-limb integration between fore- and hindlimbs correlate with degree of shared function between the two limbs? Modularity/integration studies based on morphometric and quantitative genetics tools have shown that the extent to which the limbs participate in shared functions in propulsion, support and grasping determines how much topologically similar bones of the fore- and hindlimbs co-vary in size and shape (i.e., strength and direction of integration: e.g.^6,9,11,31^). By analogy, one might predict that the bone-bone and bone-muscle connectivity networks of the fore- and hindlimb will also be similar in species with functionally convergent limbs (quantified by the proportion of time spent in shared activities such as locomotion) compared to species in which these limbs are functionally divergent, as is particularly the case in bipedal humans (i.e., *shared function leads to increased integration*). In contrast to morphometric integration analyses, our network analyses do not support this prediction. At the musculoskeletal level, *Homo sapiens* and *Pan paniscus* fore-hindlimb similarity is very close (FL/HL-ARD 5.2% and 5.3%, respectively), while *Pan paniscus* forelimb-hindlimb disparity is higher (FL/HL-ARD 8.1%) (SI1 Table 4). As noted above, at the skeletal level, the three species have the same topological organization in their forelimbs and hindlimbs, respectively, thus their forelimb-hindlimb similarity is the same. This might suggest that there is not a clear division between humans and *Pan* regarding this feature, and that each species followed a different evolutionary trajectory, which in this context lead to humans and bonobos having a higher inter-limb similarity by convergence. Alternatively, our results may indicate that similar developmental constraints or a shared developmental architecture (i.e., pleiotropy) (which, contrary to functional divergence, would tend to increase similarity between the two limbs) are strong enough to limit the extent to which each limb is modular, in terms of bone-bone and bone-muscle connectivity.

These results show that new quantitative network approaches to study integration and the constraints of evolution, including using network theory to study morphological modularity and both intra- and inter-limb integration, can complement more traditional methods such as morphometrics. Such an integrative approach can provide a more comprehensive understanding of the features that define the hominin lineage, including the evolution of uniquely human traits related to manual dexterity and bipedalism. We hope this paper will pave the way for such future works on the musculoskeletal network organization of the limbs of other primates, mammals and tetrapods.

## Materials and Methods

### Anatomical datasets

Anatomical network matrices (SI2) were constructed using information about the gross anatomy of *Homo sapiens*, *Pan troglodytes*, and *Pan paniscus* gathered from our previous works on these taxa, which were based on the dissection of several specimens of each species plus an extensive literature review of anatomical descriptions^17–20^. A comparative context is essential for establishing homologies among the muscular structures of these taxa, and for using an informed, coherent muscle nomenclature across taxa, as there are sometimes discrepancies between nomenclature derived from modern human anatomy and that used by researchers who have focused on non-human primates. Our samples consist of a total of 38 common chimpanzees and 11 bonobos dissected by us and other authors in the past (see text above). Having large samples of carefully dissected specimens is important to account for intraspecific variation in the presence/absence of specific muscles. For instance, regarding the presence/absence of the palmaris longus, we considered information obtained from all those dissections of common chimpanzees and bonobos that were reviewed for the present work. Using these samples, we coded a muscle as “present” in a species when it was present in more than 50% of the specimens we examined; the same criterion was used to assess attachments.

### Network Modeling

Using the above information about the gross anatomy of bonobos, common chimpanzees and modern humans, we built unweighted, undirected network models of the musculoskeletal anatomy of the fore- and hindlimbs of humans, chimps, and bonobos. These anatomical networks formalize the anatomical organization of the body as nodes connected by links. The coding of musculoskeletal networks of limbs following previous works^21,24^. Nodes represent skeletal elements (i.e., bones and cartilages) and muscles, while links represent physical joints among pairs of skeletal and muscular elements (e.g., articulations, attachments, and blending between muscles). Anatomical network models were coded as adjacency matrices, in which contacts present between two nodes are coded as 1 and the absence of contact between two nodes is coded as 0. We analyzed skeletal and muscular networks separately: skeletal networks include bones and cartilages as the nodes, connected by their articulations; muscular networks include muscles as the nodes, connected by tendinous joints and fibrous fusions among them. Adjacency matrices were saved as Excel sheets (see SI2) and analyzed in R^32^ using the package igraph^33^. It is important to note, because we included more musculoskeletal elements in our human matrices than in previous studies^21,23,24^, the network parameters and modules obtained for humans are more comprehensive, and different, from those described in those previous studies.

### Network Parameters

We compared the overall anatomical organization of each body part using six network parameters **(**Table 1**)**: number of nodes (*N*), number of connections (*K*), density of connections (*D*), average clustering coefficient (*C*), average shortest path length (*L*), and heterogeneity of connections. *N* and *K* account for the number of anatomical structures modeled, anatomical parts and pairwise relations. *D* is the number of actual connections with respect to the maximum possible. *D* is often used as a proxy for the complexity of a morphological structure, because the more connections among parts, the more functional possibilities, and more potential functional outcomes. *C* is the average of the sum of connections between all neighbors of each node with respect to the maximum possible, it measures the number of triangular loops or motifs in the network. *C* is used as a proxy for the relative amount of biological inter-dependence between three parts. *L* is the average of the minimum distance between all pairs of nodes in the network, distance is measured in number of connections, each one having unit length. *L* is used as a proxy of effective proximity (e.g., to work together) among anatomical parts. Together, *D*, *C*, and *L* measure the extent to which the networks are integrated. The higher number of interactions and their inter-dependences, the more integrated the system; and higher *D* and *C*, and lower *L*, in the network. Finally, *H* is the ratio between the standard deviation of connections per node and the mean number of connection per node, which provides an estimate of the irregularity of the network. *H* is used as a proxy of anisomerism, i.e., how non-homogeneous are the parts that compose the morphological structure in their number of connections. Further details on the mathematical description and morphological interpretation of these parameters have been given elsewhere^14^. We used functions from the package igraph to quantify these parameters.

### Connectivity Modules

We defined the connectivity modules present within the anatomical networks using a random walk algorithm, as implemented in the function *cluster_walktrap* of igraph. The heuristics of this algorithm is that short random walks (we used random walks of 3 steps) tend to concatenate nodes within the same module^34^. This allows to find groups of nodes (modules) that are more densely connected among them than to nodes outside of the module. The overall quality of the partition identified is evaluated using the optimization function Q defined by Newman and Girvan^35^, which is commonly used to assess whether the partition identified by an algorithm is better that what is expected at random. Q is close to 0 if the number of links within modules is no better than chance; Q is closer to 1 if the modules identified deviate from what is expected for a random network. According to Newman and Girvan’s observations, Q values in strongly modular networks range between 0.3 and 0.7. The expected error of Q was calculated using a jackknife procedure, where every link is an independent observation. Additionally, we performed a two-sample Wilcoxon rank-sum test on the internal *vs*. external connections of every module, to estimate their statistical significance. According to the general definition of a connectivity module as a group of nodes highly connected among them and poorly connected to nodes in other groups, we expect internal connections to be significantly higher than external connections (H_0_: *K*_internal_ = *K*_external_; H_a_: *K*_internal_ > *K*_external_). If H_0_ is rejected, then the module under consideration is not expected by a random grouping of nodes. Note, however, that the accuracy of this test will depend on sample size, that is, the number of nodes in the module. As a consequence, smaller modules may render unreliable p-values.

## Electronic supplementary material

Supplementary Information

Supplementary Dataset
