# Supplementary material for: First anatomical network analysis of fore- and hindlimb musculoskeletal modularity in bonobos, common chimpanzees, and humans
Source: Sci Rep. 2018 May 2;8:6885. doi: 10.1038/s41598-018-25262-6 (PMC5931964; doi:10.1038/s41598-018-25262-6)
Supplement: Supplementary file 1 — Supplementary Information [file 41598_2018_25262_MOESM1_ESM.doc]

**First anatomical network analysis of fore- and hindlimb musculoskeletal modularity in bonobos, common chimpanzees and humans**

[Short title: Network analysis of limb modularity and evolution in chimpanzees and humans]

**Authors:** Rui Diogo^1*^, Julia L. Molnar^1^, Campbell Rolian^2^, Borja Esteve-Altava^1,3^

**Affiliations:**

^1^ Department of Anatomy, Howard University College of Medicine, USA

^2^ Department of comparative biology and experimental medicine, Faculty of Veterinary Medicine, University of Calgary, Canada

^3^ Structure & Motion Lab, Department of Comparative Biomedical Sciences, Royal Veterinary College, UK

^*^ Correspondence to: Rui Diogo, Department of Anatomy, Howard University College of Medicine, Room 1101 Numa Adams Building, 520 W Street NW, 20059 Washington, DC, USA. E-mail: rui.diogo@howard.edu. Telf: (202) 640-0982

## Musculoskeletal networks - results obtained

**Supplementary Table 1 (= Table 1 of main paper).** Network parameters of musculoskeletal network.

|  | N | K | D | C | L | H |
| --- | --- | --- | --- | --- | --- | --- |
| Homo sapiens Forelimb | 94 | 193 | 0.044 | 0.380 | 3.410 | 0.923 |
| Pan troglodytes Forelimb | 99 | 245 | 0.051 | 0.405 | 3.313 | 0.788 |
| Pan paniscus Forelimb | 95 | 217 | 0.049 | 0.423 | 3.296 | 0.845 |
| Homo sapiens Hindlimb | 91 | 207 | 0.051 | 0.378 | 3.222 | 0.909 |
| Pan troglodytes Hindlimb | 97 | 217 | 0.047 | 0.381 | 3.320 | 0.962 |
| Pan paniscus Hindlimb | 98 | 222 | 0.047 | 0.391 | 3.305 | 0.974 |

**Supplementary Table 2.** Relative differences (in %) of parameters among forelimbs.

|  | N | K | D | C | L | H | Mean | SD |
| --- | --- | --- | --- | --- | --- | --- | --- | --- |
| Homo sapiens vs. Pan troglodytes | 5.2 | 23.7 | 13.4 | 6.5 | 2.9 | 15.7 | 11.3 | 7.9 |
| Homo sapiens vs. Pan paniscus | 1.1 | 11.7 | 9.6 | 10.7 | 3.4 | 8.8 | 7.5 | 4.3 |
| Pan troglodytes vs. Pan paniscus | 4.1 | 12.1 | 3.8 | 4.2 | 0.5 | 6.9 | 5.3 | 3.9 |

**Supplementary Table 3.** Relative differences (in %) of parameters among hindlimbs.

|  | N | K | D | C | L | H | Mean | SD |
| --- | --- | --- | --- | --- | --- | --- | --- | --- |
| Homo sapiens vs. Pan troglodytes | 6.4 | 4.7 | 8.1 | 0.6 | 3.0 | 5.6 | 4.7 | 2.6 |
| Homo sapiens vs. Pan paniscus | 7.4 | 7.0 | 7.9 | 3.2 | 2.6 | 6.9 | 5.8 | 2.3 |
| Pan troglodytes vs. Pan paniscus | 1.0 | 2.3 | 0.2 | 2.6 | 0.4 | 1.3 | 1.3 | 1.0 |

**Supplementary Table 4.** Relative difference (in %) between the forelimb and hindlimb of each species.

|  | N | K | D | C | L | H | Mean | SD |
| --- | --- | --- | --- | --- | --- | --- | --- | --- |
| Homo sapiens | 3.2 | 7.0 | 13.5 | 0.3 | 5.7 | 1.4 | 5.2 | 4.8 |
| Pan troglodytes | 2.0 | 12.1 | 8.0 | 6.3 | 0.2 | 19.9 | 8.1 | 7.2 |
| Pan paniscus | 3.1 | 2.3 | 4.0 | 7.8 | 0.3 | 14.3 | 5.3 | 5.1 |

**Supplementary Table 5.** Best partitions identified using the random walk-trap algorithm.

|  | # Modules | Q value | Expected error |
| --- | --- | --- | --- |
| Homo sapiens Forelimb | 5 | 0.5795726 | 0.0297993 |
| Pan troglodytes Forelimb | 11 | 0.5391254 | 0.0317625 |
| Pan paniscus Forelimb | 7 | 0.5510098 | 0.0316175 |
| Homo sapiens Hindlimb | 9 | 0.5440150 | 0.0295011 |
| Pan troglodytes Hindlimb | 7 | 0.5678184 | 0.0290254 |
| Pan paniscus Hindlimb | 6 | 0.5618761 | 0.0270400 |

**Supplementary Table 6.** Connectivity modules identified for the forelimb of *Homo sapiens*.

| ID | p-value | Elements |
| --- | --- | --- |
| 1 | 0.00196 | occipital, sternum, ribs, vertebrae, clavicle, subclavius, pectoralis.major, pectoralis.minor, serratus.anterior, rhomboid.minor, rhomboid.major |
| 2 | 1e-05 | middle.phalanx.2, middle.phalanx.3, middle.phalanx.4, middle.phalanx.5, distal.phalanx.2, distal.phalanx.3, distal.phalanx.4, distal.phalanx.5, flexor.digitorum.superficialis, flexor.digitorum.profundus, extensor.digitorum, extensor.digiti.minimi, extensor.indicis, lumbrical.1, lumbrical.2, lumbrical.3, lumbrical.4 |
| 3 | 0 | trapezoid, trapezium, scaphoid, capitate, metacarpal.1, metacarpal.2, metacarpal.3, proximal.phalanx.1, proximal.phalanx.2, proximal.phalanx.3, distal.phalanx.1, flexor.carpi.radialis, flexor.pollicis.longus, extensor.carpi.radialis.longus, extensor.carpi.radialis.brevis, extensor.pollicis.longus, adductor.pollicis, adductor.pollicis.accessorius, flexor.brevis.profundus.2, abductor.pollicis.brevis, flexor.pollicis.brevis, opponens.pollicis, interosseus.dorsalis.1, interosseus.dorsalis.2, interosseus.dorsalis.3, interosseus.palmaris.1 |
| 4 | 5e-05 | lunate, triquetrum, pisiform, hamate, metacarpal.4, metacarpal.5, proximal.phalanx.4, proximal.phalanx.5, flexor.carpi.ulnaris, opponens.digiti.minimi, flexor.digiti.minimi.brevis, abductor.digiti.minimi, interosseus.dorsalis.4, interosseus.palmaris.2, interosseus.palmaris.3 |
| 5 | 1e-05 | scapula, humerus, radius, ulna, deltoid, supraspinatus, infraspinatus, teres.minor, teres.major, subscapularis, levator.scapulae, latissimus.dorsi, biceps.brachii, coracobrachialis, brachialis, triceps.brachii, anconeus, pronator.teres, palmaris.longus, pronator.quadratus, brachioradialis, extensor.carpi.ulnaris, supinator, abductor.pollicis.longus, extensor.pollicis.brevis |

**Supplementary Table 7.** Connectivity modules identified for the forelimb of *Pan troglodytes*.

| ID | p-value | Elements |
| --- | --- | --- |
| 1 | 0.00106 | trapezium, metacarpal.1, proximal.phalanx.1, distal.phalanx.1, adductor.pollicis, flexor.brevis.profundus.2, flexor.pollicis.brevis, opponens.pollicis, abductor.pollicis.brevis, extensor.pollicis.longus, abductor.pollicis.longus |
| 2 | 0.16804 | occipital, vertebrae, rhomboideus, levator.scapulae, levator.claviculae, latissimus.dorsi, dorsoepitrochlearis |
| 3 | 0.02433 | ribs, sternum, clavicle, serratus.anterior, subclavius, pectoralis.major, pectoralis.minor |
| 4 | 0.31959 | triquetrum, pisiform, hamate, metacarpal.5, flexor.carpi.ulnaris, flexor.digiti.minimi.brevis, opponens.digiti.minimi |
| 5 | 0.04763 | humerus, radius, ulna, triceps.brachii, brachialis, pronator.quadratus, flexor.digitorum.profundus, flexor.digitorum.superficialis, palmaris.longus, epitrochleoanconeus, pronator.teres, brachioradialis, supinator, extensor.carpi.ulnaris, anconeus, extensor.digitorum |
| 6 | 0.3255 | scaphoid, lunate, trapezoid, capitate, metacarpal.2, metacarpal.3, flexor.carpi.radialis, extensor.carpi.radialis.longus, extensor.carpi.radialis.brevis |
| 7 | 0.70666 | scapula, infraspinatus, supraspinatus, deltoideus., teres.minor, subscapularis, teres.major, biceps.brachii, coracobrachialis |
| 8 | 0.00118 | metacarpal.4, proximal.phalanx.4, middle.phalanx.4, distal.phalax.4, lumbrical.3, contrahens.digitorum.4, flexor.brevis.profundus.7, flexor.brevis.profundus.8, intermetacarpal.4 |
| 9 | 0.00074 | proximal.phalanx.5, middle.phalanx.5, distal.phalanx.5, lumbrical.4, contrahens.digitorum.5, flexor.brevis.profundus.9, abductor.digiti.minimi, extensor.digiti.minimi |
| 10 | 0.00058 | proximal.phalanx.2, middle.phalanx.2, distal.phalanx.2, lumbrical.1, flexor.brevis.profundus.3, flexor.brevis.profundus.4, intermetacarpal.1, extensor.indicis |
| 11 | 0.00032 | proximal.phalanx.3, middle.phalanx.3, distal.phalanx.3, lumbrical.2, flexor.brevis.profundus.5, flexor.brevis.profundus.6, intermetacarpal.2, intermetacarpal.3 |

**Supplementary Table 8.** Connectivity modules identified for the forelimb of *Pan paniscus*.

| ID | p-value | Elements |
| --- | --- | --- |
| 1 | 0.00015 | proximal.phalanx.2, middle.phalanx.2, distal.phalanx.2, middle.phalanx.3, distal.phalanx.3, middle.phalanx.5, distal.phalanx.5, flexor.digitorum.profundus, lumbrical.1, lumbrical.2, lumbrical.4, contrahens.digitorum.5, interosseus.dorsalis.1, interosseus.palmaris.1, extensor.digitorum, extensor.digiti.minimi, extensor.indicis |
| 2 | 0.00035 | occipital, vertebrae, ribs, sternum, clavicle, serratus.anterior, rhomboideus, levator.scapulae, levator.claviculae, subclavius, pectoralis.major, pectoralis.minor, latissimus.dorsi, dorsoepitrochlearis |
| 3 | 7e-05 | scapula, humerus, radius, ulna, infraspinatus, supraspinatus, deltoideus., teres.minor, subscapularis, teres.major, triceps.brachii, brachialis, biceps.brachii, coracobrachialis, pronator.quadratus, flexor.digitorum.superficialis, palmaris.longus, epitrochleoanconeus, pronator.teres, brachioradialis, supinator, extensor.carpi.ulnaris, anconeus |
| 4 | 0.02623 | triquetrum, pisiform, hamate, metacarpal.5, proximal.phalanx.5, flexor.carpi.ulnaris, interosseus.palmaris.3, flexor.digiti.minimi.brevis, opponens.digiti.minimi, abductor.digiti.minimi |
| 5 | 0.00803 | metacarpal.4, proximal.phalanx.4, middle.phalanx.4, distal.phalax.4, lumbrical.3, contrahens.digitorum.4, interosseus.dorsalis.4, interosseus.palmaris.2 |
| 6 | 0.02298 | scaphoid, lunate, trapezoid, capitate, metacarpal.2, metacarpal.3, proximal.phalanx.3, flexor.carpi.radialis, interosseus.dorsalis.2, interosseus.dorsalis.3, extensor.carpi.radialis.longus, extensor.carpi.radialis.brevis |
| 7 | 0.00122 | trapezium, metacarpal.1, proximal.phalanx.1, distal.phalanx.1, adductor.pollicis, flexor.brevis.profundus.2, flexor.pollicis.brevis, opponens.pollicis, abductor.pollicis.brevis, extensor.pollicis.longus, abductor.pollicis.longus |

**Supplementary Table 9.** Connectivity modules identified for the hindlimb of *Homo sapiens*.

| ID | p-value | Elements |
| --- | --- | --- |
| 1 | 6e-05 | navicular, cuboid, medial.cuneiform, intermediate.cuneiform, lateral.cuneiform, metatarsal.1, metatarsal.2, metatarsal.4, proximal.phalanx.2, fibularis.longus, tibialis.anterior, tiblialis.posterior, lumbrical.1, flexor.hallucis.brevis, adductor.hallucis, dorsal.interosseus.1, dorsal.interosseus.2 |
| 2 | 0 | hip.bone, femur, patella, tibia, gluteus.maximus, gluteus.medius, gluteus.minimus, tensor.fasciae.latae, obturator.internus, obturator.externus, gemellus.superior, gemellus.inferior, iliopsoas, quadratus.femoris, pectineus, sartorius, rectus.femoris, vastus.lateralis, vastus.intermedius, vastus.medialis, adductor.brevis, adductor.magnus, adductor.longus, biceps.femoris, semitendinosus, semimembranosus, gracilis, popliteus |
| 3 | 0.25971 | fibula, calcaneus, talus, proximal.phalanx.1, plantaris, gastrocnemius, soleus, extensor.hallucis.brevis, abductor.hallucis |
| 4 | 8e-05 | middle.phalanx.2, middle.phalanx.3, middle.phalanx.4, middle.phalanx.5, distal.phalanx.2, distal.phalanx.3, distal.phalanx.4, distal.phalanx.5, extensor.digitorum.longus, flexor.digitorum.longus, extensor.digitorum.brevis, flexor.digitorum.brevis, quadratus.plantae, lumbrical.4 |
| 5 | 0.05589 | metatarsal.5, proximal.phalanx.5, fibularis.brevis, fibularis.tertius, abductor.digiti.minimi, flexor.digiti.minimi.brevis, plantar.interosseus.3 |
| 6 | 0.11717 | metatarsal.3, proximal.phalanx.3, lumbrical.2, plantar.interosseus.1, dorsal.interosseus.3 |
| 7 | 0.1844 | ribs, vertebrae, quadratus.lumborum, piriformis |
| 8 | 0.69146 | proximal.phalanx.4, lumbrical.3, plantar.interosseus.2, dorsal.interosseus.4 |
| 9 | 0.25249 | distal.phalanx.1, extensor.hallucis.longus, flexor.hallucis.longus |

**Supplementary Table 10.** Connectivity modules identified for the hindlimb of *Pan troglodytes*.

| ID | p-value | Elements |
| --- | --- | --- |
| 1 | 0.00017 | navicular, cuboid, medial.cuneiform, intermediate.cuneiform, lateral.cuneiform, metatarsal.1, metatarsal.2, proximal.phalanx.2, fibularis.longus, tibialis.anterior, tiblialis.posterior, opponens.hallucis, lumbrical.1, flexor.hallucis.brevis, adductor.hallucis, plantar.interosseus.1, dorsal.interosseus.1 |
| 2 | 0.00868 | metatarsal.3, metatarsal.4, proximal.phalanx.3, proximal.phalanx.4, lumbrical.2, lumbrical.3, plantar.interosseus.2, dorsal.interosseus.2, dorsal.interosseus.3, dorsal.interosseus.4, contrahens.pedis.4 |
| 3 | 0.04554 | fibula, calcaneus, talus, proximal.phalanx.1, distal.phalanx.1, plantaris, gastrocnemius, soleus, extensor.hallucis.longus, flexor.hallucis.longus, extensor.hallucis.brevis, abductor.hallucis |
| 4 | 0 | hip.bone, femur, patella, tibia, gluteus.medius, gluteus.minimus, ischiofemoralis, tensor.fasciae.latae, obturator.internus, obturator.externus, gemellus.superior, gemellus.inferior, quadratus.femoris, pectineus, sartorius, rectus.femoris, vastus.lateralis, vastus.intermedius, vastus.medialis, adductor.brevis, adductor.magnus, adductor.longus, adductor.minimus, biceps.femoris, semitendinosus, semimembranosus, gracilis, popliteus |
| 5 | 0.00013 | middle.phalanx.2, middle.phalanx.3, middle.phalanx.4, middle.phalanx.5, distal.phalanx.2, distal.phalanx.3, distal.phalanx.4, distal.phalanx.5, extensor.digitorum.longus, flexor.digitorum.longus, extensor.digitorum.brevis, flexor.digitorum.brevis, quadratus.plantae, lumbrical.4 |
| 6 | 0.03602 | metatarsal.5, proximal.phalanx.5, fibularis.tertius, abductor.digiti.minimi, flexor.digiti.minimi.brevis, plantar.interosseus.3, contrahens.pedis.5, opponens.digiti.minimi |
| 7 | 0.1947 | ribs, vertebrae, quadratus.lumborum, gluteus.maximus, piriformis, iliopsoas, psoas.minor |

**Supplementary Table 11.** Connectivity modules identified for the hindlimb of *Pan paniscus*.

| ID | p-value | Elements |
| --- | --- | --- |
| 1 | 0 | navicular, cuboid, medial.cuneiform, intermediate.cuneiform, lateral.cuneiform, metatarsal.1, metatarsal.2, metatarsal.3, metatarsal.4, proximal.phalanx.2, proximal.phalanx.3, proximal.phalanx.4, fibularis.longus, tibialis.anterior, tiblialis.posterior, opponens.hallucis, lumbrical.1, lumbrical.2, lumbrical.3, flexor.hallucis.brevis, adductor.hallucis, plantar.interosseus.1, plantar.interosseus.2, dorsal.interosseus.1, dorsal.interosseus.2, dorsal.interosseus.3, dorsal.interosseus.4, contrahens.pedis.4 |
| 2 | 0.03855 | fibula, calcaneus, talus, proximal.phalanx.1, distal.phalanx.1, plantaris, gastrocnemius, soleus, extensor.hallucis.longus, flexor.hallucis.longus, extensor.hallucis.brevis, abductor.hallucis |
| 3 | 0 | hip.bone, femur, patella, tibia, gluteus.medius, gluteus.minimus, scansorius, ischiofemoralis, tensor.fasciae.latae, obturator.internus, obturator.externus, gemellus.superior, gemellus.inferior, quadratus.femoris, pectineus, sartorius, rectus.femoris, vastus.lateralis, vastus.intermedius, vastus.medialis, adductor.brevis, adductor.magnus, adductor.longus, adductor.minimus, biceps.femoris, semitendinosus, semimembranosus, gracilis, popliteus |
| 4 | 0.00013 | middle.phalanx.2, middle.phalanx.3, middle.phalanx.4, middle.phalanx.5, distal.phalanx.2, distal.phalanx.3, distal.phalanx.4, distal.phalanx.5, extensor.digitorum.longus, flexor.digitorum.longus, extensor.digitorum.brevis, flexor.digitorum.brevis, quadratus.plantae, lumbrical.4 |
| 5 | 0.03602 | metatarsal.5, proximal.phalanx.5, fibularis.tertius, abductor.digiti.minimi, flexor.digiti.minimi.brevis, plantar.interosseus.3, contrahens.pedis.5, opponens.digiti.minimi |
| 6 | 0.1947 | ribs, vertebrae, quadratus.lumborum, gluteus.maximus, piriformis, iliopsoas, psoas.minor |

## Skeletal networks

**Supplementary Table 12.** Network parameters for skeletal network.

|  | N | K | D | C | L | H |
| --- | --- | --- | --- | --- | --- | --- |
| Homo sapiens Forelimb | 36 | 48 | 0.076 | 0.200 | 5.060 | 0.567 |
| Pan troglodytes Forelimb | 36 | 48 | 0.076 | 0.200 | 5.060 | 0.567 |
| Pan paniscus Forelimb | 36 | 48 | 0.076 | 0.200 | 5.060 | 0.567 |
| Homo sapiens Hindlimb | 33 | 43 | 0.081 | 0.183 | 4.695 | 0.551 |
| Pan troglodytes Hindlimb | 33 | 43 | 0.081 | 0.183 | 4.695 | 0.551 |
| Pan paniscus Hindlimb | 33 | 43 | 0.081 | 0.183 | 4.695 | 0.551 |

**Supplementary Table 13.** Relative differences (in %) of parameters among forelimb skeletons.

|  | N | K | D | C | L | H | Mean | SD |
| --- | --- | --- | --- | --- | --- | --- | --- | --- |
| Homo sapiens vs. Pan troglodytes | 0 | 0 | 0 | 0 | 0 | 0 | 0 | 0 |
| Homo sapiens vs. Pan paniscus | 0 | 0 | 0 | 0 | 0 | 0 | 0 | 0 |
| Pan troglodytes vs. Pan paniscus | 0 | 0 | 0 | 0 | 0 | 0 | 0 | 0 |

**Supplementary Table 14.** Relative differences (in %) of parameters among hindlimb skeletons.

|  | N | K | D | C | L | H | Mean | SD |
| --- | --- | --- | --- | --- | --- | --- | --- | --- |
| Homo sapiens vs. Pan troglodytes | 0 | 0 | 0 | 0 | 0 | 0 | 0 | 0 |
| Homo sapiens vs. Pan paniscus | 0 | 0 | 0 | 0 | 0 | 0 | 0 | 0 |
| Pan troglodytes vs. Pan paniscus | 0 | 0 | 0 | 0 | 0 | 0 | 0 | 0 |

**Supplementary Table 15.** Relative difference (in %) between the forelimb and hindlimb skeletons.

|  | N | K | D | C | L | H | Mean | SD |
| --- | --- | --- | --- | --- | --- | --- | --- | --- |
| Homo sapiens | 8.7 | 11 | 6.7 | 8.7 | 7.5 | 2.9 | 7.6 | 2.7 |
| Pan troglodytes | 8.7 | 11 | 6.7 | 8.7 | 7.5 | 2.9 | 7.6 | 2.7 |
| Pan paniscus | 8.7 | 11 | 6.7 | 8.7 | 7.5 | 2.9 | 7.6 | 2.7 |

**Supplementary Table 16.** Best partitions identified using the random walk-trap algorithm.

|  | # Modules | Q value | Expected error |
| --- | --- | --- | --- |
| Homo sapiens Forelimb | 9 | 0.5000000 | 0.0797776 |
| Pan troglodytes Forelimb | 9 | 0.5000000 | 0.0797776 |
| Pan paniscus Forelimb | 9 | 0.5000000 | 0.0797776 |
| Homo sapiens Hindlimb | 8 | 0.5359654 | 0.0809061 |
| Pan troglodytes Hindlimb | 8 | 0.5359654 | 0.0809061 |
| Pan paniscus Hindlimb | 8 | 0.5359654 | 0.0809061 |

**Supplementary Table 17.** Connectivity modules identified for the forelimb skeleton of *Homo sapiens*.

| ID | p-value | Elements |
| --- | --- | --- |
| 1 | 3e-05 | trapezoid, trapezium, scaphoid, lunate, triquetrum, pisiform, hamate, capitate, metacarpal.2, metacarpal.3, metacarpal.4, metacarpal.5 |
| 2 | 0.04318 | sternum, ribs, clavicle, scapula |
| 3 | 0.07865 | proximal.phalanx.5, middle.phalanx.5, distal.phalanx.5 |
| 4 | 0.07865 | metacarpal.1, proximal.phalanx.1, distal.phalanx.1 |
| 5 | 0.07865 | proximal.phalanx.2, middle.phalanx.2, distal.phalanx.2 |
| 6 | 0.07865 | proximal.phalanx.4, middle.phalanx.4, distal.phalanx.4 |
| 7 | 0.07865 | proximal.phalanx.3, middle.phalanx.3, distal.phalanx.3 |
| 8 | 0.09835 | humerus, radius, ulna |
| 9 | 0.30854 | occipital, vertebrae |

**Supplementary Table 18.** Connectivity modules identified for the forelimb skeleton of *Pan troglodytes*.

| ID | p-value | Elements |
| --- | --- | --- |
| 1 | 3e-05 | scaphoid, lunate, triquetrum, pisiform, trapezium, trapezoid, capitate, hamate, metacarpal.2, metacarpal.3, metacarpal.4, metacarpal.5 |
| 2 | 0.04318 | ribs, sternum, scapula, clavicle |
| 3 | 0.07865 | proximal.phalanx.5, middle.phalanx.5, distal.phalanx.5 |
| 4 | 0.07865 | metacarpal.1, proximal.phalanx.1, distal.phalanx.1 |
| 5 | 0.07865 | proximal.phalanx.2, middle.phalanx.2, distal.phalanx.2 |
| 6 | 0.07865 | proximal.phalanx.4, middle.phalanx.4, distal.phalax.4 |
| 7 | 0.07865 | proximal.phalanx.3, middle.phalanx.3, distal.phalanx.3 |
| 8 | 0.09835 | humerus, radius, ulna |
| 9 | 0.30854 | occipital, vertebrae |

**Supplementary Table 19.** Connectivity modules identified for the forelimb skeleton of *Pan paniscus*.

| ID | p-value | Elements |
| --- | --- | --- |
| 1 | 3e-05 | scaphoid, lunate, triquetrum, pisiform, trapezium, trapezoid, capitate, hamate, metacarpal.2, metacarpal.3, metacarpal.4, metacarpal.5 |
| 2 | 0.04318 | ribs, sternum, scapula, clavicle |
| 3 | 0.07865 | proximal.phalanx.5, middle.phalanx.5, distal.phalanx.5 |
| 4 | 0.07865 | metacarpal.1, proximal.phalanx.1, distal.phalanx.1 |
| 5 | 0.07865 | proximal.phalanx.2, middle.phalanx.2, distal.phalanx.2 |
| 6 | 0.07865 | proximal.phalanx.4, middle.phalanx.4, distal.phalax.4 |
| 7 | 0.07865 | proximal.phalanx.3, middle.phalanx.3, distal.phalanx.3 |
| 8 | 0.09835 | humerus, radius, ulna |
| 9 | 0.30854 | occipital, vertebrae |

**Supplementary Table 20.** Connectivity modules identified for the hindlimb skeleton of *Homo sapiens*.

| ID | p-value | Elements |
| --- | --- | --- |
| 1 | 0.00744 | femur, patella, tibia, fibula, calcaneus, talus |
| 2 | 0.00012 | navicular, cuboid, medial.cuneiform, intermediate.cuneiform, lateral.cuneiform, metatarsal.2, metatarsal.3, metatarsal.4, metatarsal.5 |
| 3 | 0.07865 | ribs, vertebrae, hip.bone |
| 4 | 0.07865 | proximal.phalanx.5, middle.phalanx.5, distal.phalanx.5 |
| 5 | 0.07865 | metatarsal.1, proximal.phalanx.1, distal.phalanx.1 |
| 6 | 0.07865 | proximal.phalanx.2, middle.phalanx.2, distal.phalanx.2 |
| 7 | 0.07865 | proximal.phalanx.4, middle.phalanx.4, distal.phalanx.4 |
| 8 | 0.07865 | proximal.phalanx.3, middle.phalanx.3, distal.phalanx.3 |

**Supplementary Table 21.** Connectivity modules identified for the hindlimb skeleton of *Pan troglodytes*.

| ID | p-value | Elements |
| --- | --- | --- |
| 1 | 0.00744 | femur, patella, tibia, fibula, calcaneus, talus |
| 2 | 0.00012 | navicular, cuboid, medial.cuneiform, intermediate.cuneiform, lateral.cuneiform, metatarsal.2, metatarsal.3, metatarsal.4, metatarsal.5 |
| 3 | 0.07865 | ribs, vertebrae, hip.bone |
| 4 | 0.07865 | proximal.phalanx.5, middle.phalanx.5, distal.phalanx.5 |
| 5 | 0.07865 | metatarsal.1, proximal.phalanx.1, distal.phalanx.1 |
| 6 | 0.07865 | proximal.phalanx.2, middle.phalanx.2, distal.phalanx.2 |
| 7 | 0.07865 | proximal.phalanx.4, middle.phalanx.4, distal.phalanx.4 |
| 8 | 0.07865 | proximal.phalanx.3, middle.phalanx.3, distal.phalanx.3 |

**Supplementary Table 22.** Connectivity modules identified for the hindlimb skeleton of *Pan paniscus*.

| ID | p-value | Elements |
| --- | --- | --- |
| 1 | 0.00744 | femur, patella, tibia, fibula, calcaneus, talus |
| 2 | 0.00012 | navicular, cuboid, medial.cuneiform, intermediate.cuneiform, lateral.cuneiform, metatarsal.2, metatarsal.3, metatarsal.4, metatarsal.5 |
| 3 | 0.07865 | ribs, vertebrae, hip.bone |
| 4 | 0.07865 | proximal.phalanx.5, middle.phalanx.5, distal.phalanx.5 |
| 5 | 0.07865 | metatarsal.1, proximal.phalanx.1, distal.phalanx.1 |
| 6 | 0.07865 | proximal.phalanx.2, middle.phalanx.2, distal.phalanx.2 |
| 7 | 0.07865 | proximal.phalanx.4, middle.phalanx.4, distal.phalanx.4 |
| 8 | 0.07865 | proximal.phalanx.3, middle.phalanx.3, distal.phalanx.3 |
